# Pituitary apoplexy and COVID-19 vaccination: a case report and literature review

**DOI:** 10.3389/fendo.2022.1035482

**Published:** 2022-11-17

**Authors:** Ludovica Aliberti, Irene Gagliardi, Roberta Rizzo, Daria Bortolotti, Giovanna Schiuma, Paola Franceschetti, Roberta Gafà, Luca Borgatti, Michele A. Cavallo, Maria C. Zatelli, Maria R. Ambrosio

**Affiliations:** ^1^ Department of Medical Sciences, Section of Endocrinology and Internal Medicine, University of Ferrara, Ferrara, Italy; ^2^ Department of Chemical, Pharmaceutical and Agricultural Sciences, University of Ferrara, Ferrara, Italy; ^3^ Unit of Endocrinology and Metabolic Diseases – Oncology and Specialty Medicines Department, Azienda Ospedaliero Universitaria di Ferrara, Ferrara, Italy; ^4^ Department of Translational Medicine, University of Ferrara, Ferrara, Italy; ^5^ Neuroradiology Unit, Department of Neuroscience and Rehabilitation, Azienda Ospedaliera Universitaria, Arcispedale S. Anna, Ferrara, Italy; ^6^ Neurosurgery Department, University Hospital S. Anna, Ferrara, Italy

**Keywords:** COVID - 19, SARS – CoV – 2, pituitary apoplexy, vaccine-induced thrombotic thrombocytopenia (VITT), autoimmunity

## Abstract

A 50-year-old man was admitted to our hospital for vomit, nausea, diplopia, and headache resistant to analgesic drugs. Symptoms started the day after his third COVID-19 mRNA vaccine (Moderna) whereas SARS-CoV-2 nasal swab was negative. Pituitary MRI showed recent bleeding in macroadenoma, consistent with pituitary apoplexy. Adverse Drug Reaction was reported to AIFA (Italian Medicines Agency).A stress dexamethasone dose was administered due to the risk of adrenal insufficiency and to reduce oedema. Biochemistry showed secondary hypogonadism; inflammatory markers were elevated as well as white blood cells count, fibrinogen and D-dimer. Pituitary tumour transsphenoidal resection was performed and pathology report was consistent with pituitary adenoma with focal haemorrhage and necrosis; we found immunohistochemical evidence for SARS-CoV-2 proteins next to pituitary capillaries, in the presence of an evident lymphocyte infiltrate.Few cases of pituitary apoplexy after COVID-19 vaccination and infection have been reported. Several hypotheses have been suggested to explain this clinical picture, including cross-reactivity between SARS-CoV-2 and pituitary proteins, COVID-19-associated coagulopathy, infection-driven acutely increased pituitary blood demand, anti-Platelet Factor 4/heparin antibodies development after vaccine administration. Ours is the first case of SARS-CoV-2 evidence in pituitary tissue, suggesting that endothelial infection of pituitary capillaries could be present before vaccination, possibly due to a previous asymptomatic SARS-CoV-2 infection. Our case underlines that SARS-CoV-2 can associate with apoplexy by penetrating the central nervous system, even in cases of negative nasal swab. Patients with pituitary tumours may develop pituitary apoplexy after exposure to SARS-CoV-2, therefore clinicians should be aware of this risk.

## Introduction

Pituitary apoplexy is a rare endocrine emergency caused by haemorrhage and/or infarction within the pituitary gland that may require decompressive surgery and specific treatment (1). Pituitary apoplexy most often involves a pituitary adenoma and can occur either spontaneously or due to a stressful trigger (1). The immediate medical management includes supportive measures to ensure haemodynamic stability, fluid and electrolyte balance. Prompt corticosteroid replacement should be started in patients who are haemodynamically unstable or who have other signs or symptoms suggestive of hypoadrenalism (2, 3). In adults, intravenous hydrocortisone treatment is the most appropriate strategy, whereas dexamethasone is not favoured as glucocorticoid replacement, although it may be used to reduce oedema within the pituitary gland (2, 3). Patients with severe neuro-ophthalmic signs, such as severely reduced visual acuity, severe and persistent or deteriorating visual field defects or deteriorating level of consciousness should be considered for surgical management (2, 3). Risk factors for apoplexy are summarized in **Table 1** (2). There are few reports of pituitary adenoma apoplexy after viral infection, such as haemorrhagic fever, 2003 Severe Acute Respiratory Syndrome (SARS) epidemic, influenza A and, recently, SARS-CoV-2 (1, 2, 4–6). Less is known on the relationship between pituitary apoplexy and SARS-CoV-2 vaccine. Here, we report the case of a man hospitalized for severe headache after SARS-CoV-2 vaccine with a subsequent diagnosis of pituitary apoplexy in macroadenoma, not previously diagnosed. In addition, the present study reports for the first time immunohistochemical evidence for SARS-CoV-2 proteins in pituitary tissues.

**Table 1:** Risk factors for pituitary apoplexy.

Risk factors for pituitary apoplexy	
Pregnancy and post-partum state	Head trauma
Coagulopathy, thrombocytopenia, anticoagulation	Oestrogen therapy
Radiotherapy	Acute increase in blood flow: physical activity, systemic hypertension
Pituitary stimulation: provocative pituitary tests, specially TRH, GnRH analogues	Vascular flux reduction: surgery (specially cardiac surgery) post spinal anaesthesia
Diabetes mellitus	Sickle cell anaemia
Lymphocytic leukemia	Dopamine agonist therapy

## Case report

**Figure 1:**
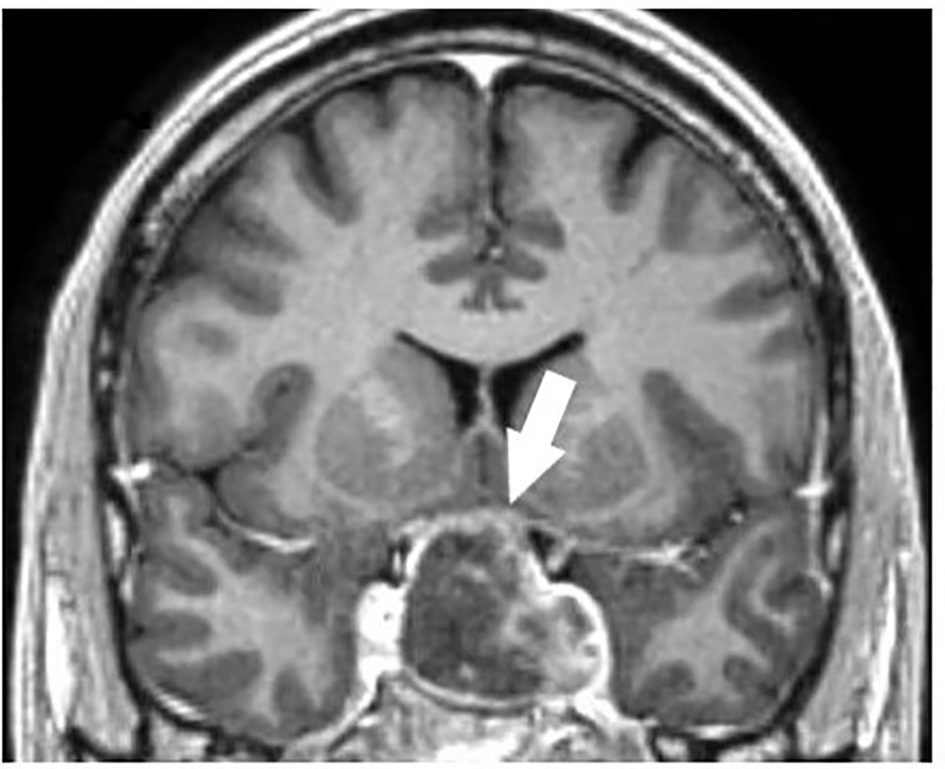
Sellar magnetic resonance imaging (MRI), confirming recent signs of haemorrhage in macroadenoma, compressing optic chiasm and extending to suprasellar cisterns and left cavernous sinus.

A 50-year-old man presented to the hospital for severe headache, vomit, nausea and diplopia that started after receiving the third COVID-19 vaccine shot (Moderna). Previously he received Pfizer-BioNTech vaccine without side effects. The day following the vaccine dose, he developed headache, unsuccessfully treated with over-the-counter analgesics at home. After the onset of nausea, vomit, and diplopia, he was admitted to the Emergency Department of Ferrara Hospital (Italy), where a cerebral computed tomography (CT) showed a sellar enlargement due to an intra and suprasellar mass, consistent with a macroadenoma. Vital signs showed hypotension and fever (37.5°C), therefore a stress dexamethasone dose was administered to prevent adrenal insufficiency symptoms and to improve oedema. At our Unit, he underwent pituitary magnetic resonance imaging (MRI), confirming recent signs of haemorrhage in a 35 x 27 x 39 mm macroadenoma, compressing the optic chiasm and extending to suprasellar cisterns and left cavernous sinus (**Figure 1**). MRI documented erosion of the floor and the back of the sella turcica, and no visible pituitary stalk. The pituitary gland was dislocated in the postero-superior right side of the mass. Radiological report was consistent with pituitary apoplexy in pituitary macroadenoma. Hypophysitis diagnosis was excluded also on the basis of the absence of typical MRI findings. Biochemistry showed normal TSH, free thyroxine (fT4) and Prolactin (PRL) levels and documented secondary hypogonadism; Adrenocorticotropic hormone (ACTH) and cortisol were not investigated since the patient was treated with corticosteroids (**Table 2**). Kidney and liver function, calcium and phosphate were within reference ranges, whereas Erythrocyte Sedimentation Rate (ESR), D-dimer and C-Reactive Protein (CRP) were increased as well as white blood count (17.71 x 10^3^/mcL with normal values < 10 x 10^3^/mcL) (**Table 2**). Sodium and potassium plasma levels were at the lower limit of the reference range (136 mmol/L and 3.9 mmol/L, respectively). Mild anaemia was present (Hb 12.5 g/dL) whereas platelets were within the low normal reference range (176 x 10^3^/mcL with normal values >150 x 10^3^/mcL) and fibrinogen was high (399 mg/dl with normal values < 300 mg/dl). SARS-CoV-2 nasal swab was repeatedly negative throughout the hospital stay. Anti-SARS-CoV-2 Immunoglobulin (Ig) G antibodies (Ab) title was high (134.81 AU/ml; normal values <10) whereas anti SARS-CoV-2 IgM Ab title was low (0.68 AU/ml; normal values < 1).

**Table 2:** Timeline with relevant data from the episode of care.

1^st^ day	2^nd^ day	4^th^ day	5^th^ day	6^th^ day
SARS-CoV- 2 Vaccine (Moderna)	Nausea, vomit, severe headache, diplopia, fever, hypotension. SARS-CoV-2 nasal swab negative	Persistence of headache (Pain VAS 8/10), diplopia and fever. SARS-CoV-2 nasal swab negative	Persistence of headache (Pain VAS 8/10) and diplopia. Fever worsening (38.5°C)	Persistence of headache (Pain VAS 6/10). SARS-CoV-2 nasal swab negative
	Admission to the Emergency Department	Admission to the Endocrinology Department	Normal visual acuity, visual field, ocular tone, and fundus. Diplopia Persistence with high grade of deviation	Endoscopic transsphenoidal resection of the pituitary tumour
	Cerebral CT: sellar enlargement caused by a intra and suprasellar mass consistent with macroadenoma	Pituitary MRI confirming the presence of pituitary macroadenoma (35x27x39 mm)	Neurological examination excluded meningism	
	Start of stress dose dexamethasone (8 mg/day) due to the risk of adrenal insufficiency and to reduce oedema	Biochemistry: TSH 0.41 μIU/mL (n.v. 0.25-4.5) fT4 7.1 pg/mL (n.v. 5.5-12) FSH 1.9 mIU/mL (n.v.1.2- 8.6) LH 0.6 mIU/mL (n.v. 1.3- 8) Test 0.29 ng/ml (n.v. 2.35-3.5) PRL 5.1 ng/mL (n.v. 5-20) ESR: 19 mm (n.v. 0-18) CRP: 1.93 mg/dl (n.v. < 0.5) D-dimer:0.59 mg/l (n.v.<0.5)	Start of morphine for pain due to headache with clinical improvement. Stress dexamethasone dose (8 mg/day)	
		Stress dose dexamethasone (8 mg/day)		

fT4, free thyroxine; FSH, Follicle-stimulating hormone; LH, Luteinizing hormone; Test, Testosterone; PRL, Prolactin; Erythrocyte Sedimentation Rate (ESR); CRP, C- Reactive Protein; nv, normal values; VAS, Visual analogue scale.

Visual field was normal, as well as visual acuity (right eye: 10/10; left eye: 9/10), ocular tone (12 mmHg) and fundus (absent papilledema). Orthoptic examination confirmed diplopia. After two days, hemodynamical parameters were stable but fever raised (38.5°C) and headache did not improve (Visual Analogues Scale: 8/10). Morphine treatment was started, followed by headache improvement. The patient underwent endoscopic transsphenoidal resection of the pituitary tumour. Pathology report was consistent with chromophobic adenoma fragments with necrotic-haemorrhagic areas and ACTH -/TSH immunophenotype -/GH -/LH -/FSH -/PRL -/ER -/CK 8-18 ± (rare cells)/Chromogranin +; Ki67: about 1-2%. Therefore, findings were consistent with non-secreting pituitary adenoma.

**Figure 2:**
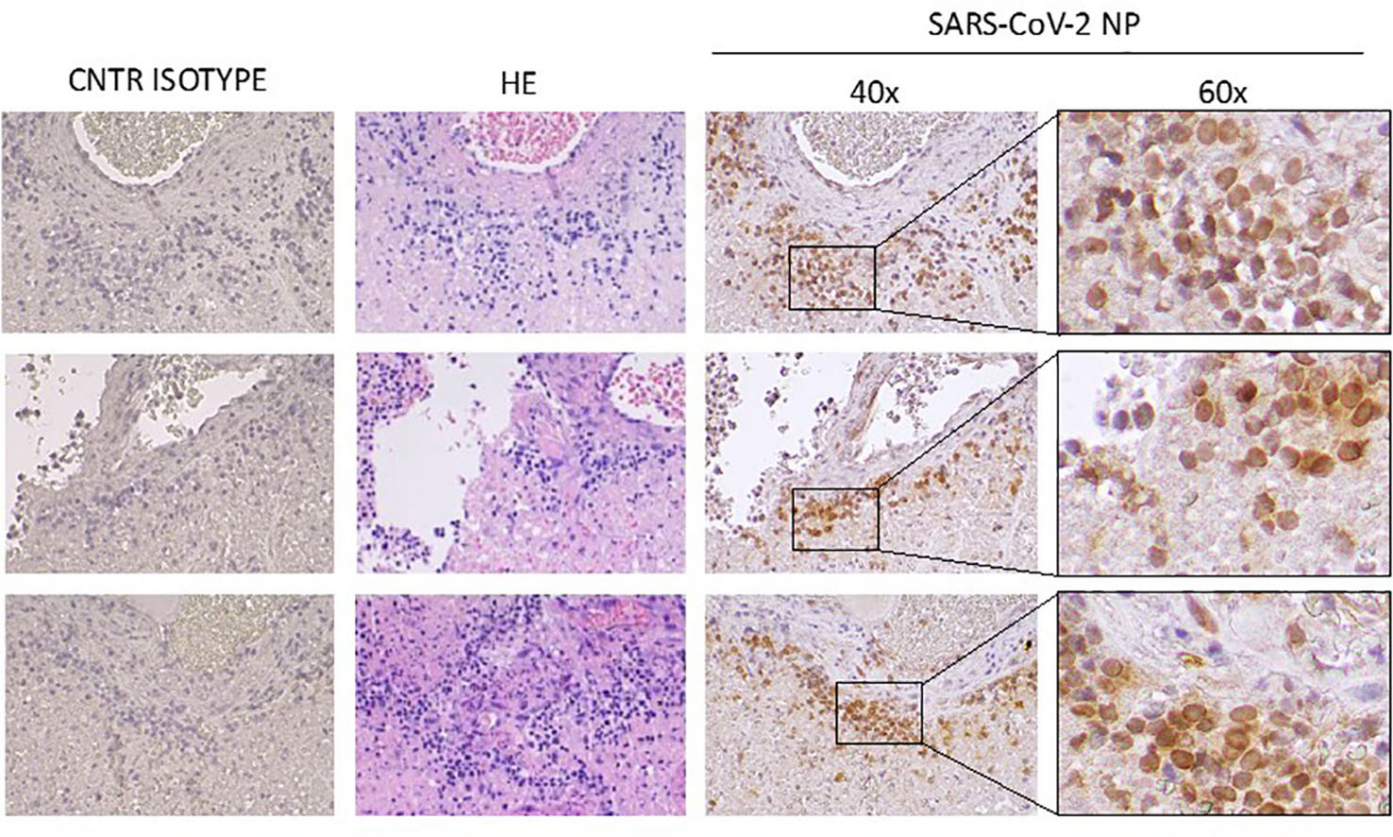
IHC staining for control isotype (CNTR ISOTYPE); Haematoxylin-Eosin (HE) and SARS-CoV-2 NP (right). Magnification: 40x and 60x.

Immunohistochemistry (IHC) was performed on 4 μm tissue slides for the detection of SARS-CoV-2 nuclear proteins (NP) (NB100-56576, Novus Biologicals, Centennial, 1:250 dilution) and counterstained with Hematoxylin-Eosin for histo-morphological analysis, as described previously (7). A control Isotype was used to define staining specificity. IHC images were analysed using QuPath software to calculate H-Score for SARS-CoV-2 NP staining. An H-Score between 0 and 300 was obtained where 300 was equal to 100% of cells strongly stained (3+). IHC analysis confirmed the presence of SARS-CoV-2 infection in correspondence of damaged tissue (**Figure 2**; H-Score 15,7 ± 9,9). Interestingly, we found SARS-CoV-2 NP expression next to pituitary vessels, in the presence of an evident lymphocyte infiltrate.

After neurosurgery, oral cortisone acetate was started (25 mg/day) as replacement therapy, and then gradually reduced until withdrawal because of adrenal function normalization as witnessed by the optimal cortisol response to low-dose ACTH test at 12 weeks after surgery.

At 4 months follow-up the patient did not present any residual pituitary adenoma, all pituitary hormones were normal, headache and diplopia disappeared. He has been submitted to clinical and biochemical periodic follow-up.

The patient had two children, did not smoke, and did not take any pharmacological treatment. He reported kidney stones 15 years before. Vaccine Adverse Drug Reaction was reported to AIFA (Italian Medicines Agency).

## Discussion

Our report describes the case of pituitary apoplexy after SARS-CoV-2 vaccine with subsequent diagnosis of macroadenoma. In addition, we report for the first time the evidence of SARS-CoV-2 nuclear proteins in pituitary tissue, suggesting that endothelial pituitary vessels infection might be present before vaccination. This aspect, together with a repeatedly negative nasal swab and the negativity of anti-SARS-CoV-2 IgM Ab title, supports the hypothesis that the patient could have experienced a previous asymptomatic SARS-CoV-2 infection. It is likely that SARS-CoV-2 persisted in the central nervous system (CNS), and that was implicated in the apoplexy onset, triggered by immunological response due to COVID-19 vaccine. Finally, the present report confirms the safety of endonasal surgery regardless of COVID-19 status, since no evidence for significant contamination of the surgical field was found.

Currently, two vaccines are authorized and recommended in Italy to prevent COVID-19: Pfizer-BioNTech vaccine and Moderna vaccine. Over the last year, Johnson & Johnson/Janssen and AstraZeneca viral vector vaccines were also used. Vaccines may lead to thrombosis and bleeding associated with the Vaccine-Induced Thrombotic Thrombocytopenia syndrome (VITT). A case of a 37-year-old woman developing a high-intensity frontal headache 5 days after vaccination with ChAdOx1-S (AstraZeneca) has been previously reported (8). In that case, brain MRI showed pituitary haemorrhagic bleeding in association with a possible 10 mm intraglandular adenoma. The patient did not have pituitary hormonal deficits at the time of observation and symptoms disappeared within 2–3 weeks with no treatment. However, a hemogram was not performed at apoplexy onset and, two months later, blood count was normal, therefore VITT could not be demonstrated (8). Another report describes the case of a 44-year-old man with fever, chills, hypotension, change in mental status and blurry vision three days after the second COVID-19 vaccine (unknown type) (9). Head MRI showed a 4.7 cm sellar and suprasellar mass with optic chiasm compression and left sphenoidal extension. Hormonal exams and clinical status suggested adrenal insufficiency therefore hydrocortisone treatment was started and transsphenoidal surgery of the pituitary mass was performed. Histology was consistent with pituitary adenoma with focal haemorrhage and necrosis. Another report concerns a 28-year-old healthy Caucasian female showing fever (38°C) for 24 h and headache for a full month after the first dose of AstraZeneca vaccine, exacerbating after the second dose. MRI showed an oval area of ~10x5 mm consistent with haemorrhage in the right half of the sella turcica (10). The last report deals with the case of a 24-year-old female with no known medical condition, presenting with frontal headache the day after receiving her second AstraZeneca vaccine dose. MRI showed pituitary apoplexy with an underlying pituitary mass, suspected for hypophysitis, measuring 1.1 × 1.6 × 1.9 cm (Anteroposterior x Transverse x Craniocaudal). Biochemistry detected secondary hypocortisolism, therefore intravenous hydrocortisone was started with symptom improvement and significant 50% reduction in size of the pituitary mass. Authors concluded that the patient might have developed an exaggerated immunological response after the vaccine with a possible hypophysitis leading to a pituitary apoplexy (11).

An hypophysitis case after the second dose of Moderna vaccine presented with frontal headache, nausea, vomit, and abdominal pain (12). The patient was treated with high-dose steroids and, after 1 month, follow-up pituitary MRI revealed markedly reduced pituitary with an almost empty sella. However, differential diagnosis between hypophysitis and pituitary apoplexy is complex and pituitary biopsy and histopathological analysis is rarely performed.

Finally, few cases of pituitary apoplexy during SARS-CoV-2 infection are described. All of them presented with severe headache and with positive nasal swabs for SARS-CoV-2 infection (13–19). According to our report and literature data, symptoms related to pituitary apoplexy may occur 2-5 days after COVID-19 vaccine. The most frequently reported symptom is a high-intensity headache, suggesting that an MRI should be performed to look for an adenoma when headache does not improve after glucocorticoids or analgesic treatment. Even though few cases are described, male patients seem to present more frequently with adrenal insufficiency and pituitary macroadenoma with suprasellar invasion. By contrast, female patients have smaller adenomas, more frequently treated in a conservative manner, or may develop a hypophysitis. These differences may depend on the possible delay in the diagnosis induced by the gender differences in symptom presentation/reporting. It has been recently described that non-functioning pituitary adenomas (NFPA) > 1 cm are more frequent in males than females and it is known that macroprolactinomas occur mostly in men (20, 21). However, the existing literature does not provide evidence for a significant association between COVID-19 and pituitary apoplexy, neither for gender-related differences.

Autoimmunity and VITT may be the causes of pituitary apoplexy. The immune cross-reactivity triggered by the similarity between vaccine components and specific human proteins (molecular mimicry) could induce the immune system to attack similar proteins (22–26). However, only a minority of vaccinated subjects develops autoimmune phenomena, indicating a genetic predisposition to vaccine-induced autoimmunity. Some reports suggest that the SARS-CoV-2 virus can reach the brainstem and neural tissues *via* two potential routes: either by hematogenous spread or through the nasopharyngeal epithelium and the olfactory nerve. Moreover, viral tropism for neural tissues is mediated by cerebral vascular endothelial expression of Angiotensin Converting Enzyme 2 (ACE2) receptors, which are binding sites for the virus (5, 6). Prothrombotic coagulopathy has been recorded in COVID-19 patients and a marked complement activation *via* lectin and alternative coagulation pathway was observed, with consequent consumption coagulopathy and VITT. These aspects, in combination with overstimulation of the pituitary gland in the settings of an acute infection, couldprecipitate pituitary infarction and/or haemorrhage (1, 27, 28).

In addition, infectious agents may trigger autoimmune mechanisms and cause autoimmune diseases (19–23). Recently, Frara et al. and Kamel et al. showed that SARS-CoV-2 may cause pituitary apoplexy in the context of pre-existing macroadenomas (1, 6). Previously, Tariq et al. reported an association between influenza A infection and pituitary tumour apoplexy (4). Even though a causative link could not be demonstrated, influenza infections have been documented to increase haemorrhagic risk in multiple organs (4). Other reports describe a possible relationship between viral infections (i.e., haemorrhagic fever, 2003 SARS epidemic) and direct pituitary vascular damage with ischemic and haemorrhagic signs as well as necrosis at post-mortem evaluation (4–6).

VITT is caused by IgG antibodies that recognize platelet factor 4 (PF4) bound to platelets. Consequently, platelet activation occurs, stimulating the coagulation system and causing clinically significant thromboembolic complications (24, 25). Thrombotic events are related not only to adenovirus vector vaccine but also to mRNA vaccine. Welsh et al. reported 15 and 13 cases of thrombocytopenia among >16 million doses of COVID-19 vaccine (Moderna) and >18 million doses of COVID-19 vaccine (Pfizer-BioNTech), respectively (29). In addition, vaccine adjuvants could increase vaccine immunogenicity by triggering the nucleotide-binding domain leucine-rich repeat containing (NLR) pyrin domain containing 3 (NLRP3) inflammasome (29, 30). Moreover, mRNA vaccines contain lipid nanoparticles (LNPs) to protect mRNA from degradation. LNPs could trigger inflammatory responses, characterized by massive neutrophil infiltration, activation of inflammatory pathways, and production of various inflammatory cytokines in mouse (30). Finally, mRNA itself may trigger inflammation and immunity (31, 32). In our case, fibrinogen levels were high whereas platelet count was within the low reference range. However, because of the immediate start of corticosteroid treatment, we did not analyse PF4 antibody levels.

Patients with pituitary apoplexy who are haemodynamically unstable or with signs/symptoms of adrenal insufficiency should start empirical steroid therapy. In adults, hydrocortisone 100–200 mg as an intravenous bolus is appropriate, followed either by 2–4 mg/hour continuous intravenous infusion or by 50–100 mg six hourly intramuscular injection. Dexamethasone is not favoured as glucocorticoid replacement, although it may be used to reduce oedema as part of a nonsurgical strategy for the treatment of pituitary tumour apoplexy (2, 3). Systemic dexamethasone therapy also improves clinical outcome and reduces mortality in hospitalized patients with COVID-19 infection who require oxygen, presumably by mitigating COVID-19-induced systemic inflammatory response (33).

From the patient’s perspective, adrenal insufficiency, headache, and diplopia were experienced during the acute disease. The patient reported that pain was unbearable and that he thought he would have died. On the contrary, symptoms gradually disappeared during follow up thanks to a prompt and appropriate management of pituitary apoplexy. The patient was very satisfied of the outcome, thankful to all the hospital staff and happy to provide consent for the publication of his medical history.

COVID-19 infection clinical manifestations may be worsened by concomitant hypopituitarism that must be promptly recognized and treated. Finally, a limit of our study is that we did not research for plasma SARS-CoV-2 in the patient.

## Conclusions

The possibility of pituitary apoplexy following COVID-19 vaccination should be considered. Patients with pituitary tumours may develop pituitary apoplexy after exposure to SARS-CoV-2, therefore clinicians should be aware of this risk, especially in cases of severe headache, investigating pituitary function.

## Data availability statement

The raw data supporting the conclusions of this article will be made available by the authors, without undue reservation.

## Ethics statement

Written informed consent was obtained from the individual for the publication of any potentially identifiable images or data included in this article.

## Author contributions

LA, IG, MCZ and MA analysed and interpreted the patient data. LA, IG and MZ were the major contributor in writing the manuscript. IG, DB, RR, GS, PF, RG, LB, MC and MA revised critically the manuscript. MA ideated the article. All authors contributed to the article and approved the submitted version.

## Funding

This research was in part supported by funds of the University of Ferrara (FAR 2019, 2020, 2021) and by PRIN 2017 (grant number: PRIN 2017Z3N3YC).

## Conflict of interest

The authors declare that the research was conducted in the absence of any commercial or financial relationships that could be construed as a potential conflict of interest.

## Publisher’s note

All claims expressed in this article are solely those of the authors and do not necessarily represent those of their affiliated organizations, or those of the publisher, the editors and the reviewers. Any product that may be evaluated in this article, or claim that may be made by its manufacturer, is not guaranteed or endorsed by the publisher.
